# Tick-Borne Encephalitis: A Differential Pattern of Intrathecal Humoral Immune Response and Inflammatory Cell Composition Compared with Other Viral CNS Infections

**DOI:** 10.3390/cells9102169

**Published:** 2020-09-25

**Authors:** Makbule Senel, Daniel Rapp, Benjamin Mayer, Sarah Jesse, Sigurd D. Süssmuth, Markus Otto, Jan Lewerenz, Hayrettin Tumani

**Affiliations:** 1 Department of Neurology, University of Ulm, 89081 Ulm, Germany; daniel.rapp@uni-ulm.de (D.R.); sarah.jesse@uni-ulm.de (S.J.); sigurd.suessmuth@uni-ulm.de (S.D.S.); markus.otto@uni-ulm.de (M.O.); jan.lewerenz@uni-ulm.de (J.L.); hayrettin.tumani@uni-ulm.de (H.T.); 2 Institute of Epidemiology and Medical Biometry, Ulm University, 89075 Ulm, Germany; benjamin.mayer@uni-ulm.de; 3 Speciality Clinic of Neurology Dietenbronn, 88477 Schwendi, Germany

**Keywords:** TBE, tick-borne encephalitis, TBEV, HSV, VZV, viral, meningitis, encephalitis

## Abstract

To investigate whether and how cerebrospinal fluid (CSF) findings can contribute to distinguish tick-borne encephalitis (TBE) from herpes simplex virus (HSV) and varicella zoster virus (VZV) induced central nervous system (CNS) infections (HSV-I, VZV-I). Chart review and identification of TBE, HSV- I, and VZV-I was carried out, fulfilling the following criteria: (1) clinical signs of encephalitis and/or meningitis, (2) complete CSF analysis and confirmed viral etiology by either PCR or antibody testing in CSF, (3) hospitalized patients, and (4) available brain magnetic resonance imaging (MRI). Fifty-nine patients with 118 CSF/serum pairs were included. These comprised 21 with TBE (35 CSF/serum pairs), 20 (40 CSF/serum pairs) with HSV-I, and 18 (43 CSF/serum pairs) with VZV-I. In contrast to HSV-I and VZV-I, CSF cell differentiation in TBE showed more often an increased (>20%) proportion of granulocytes (*p* < 0.01) and a more frequent quantitative intrathecal IgM synthesis (*p* = 0.001 and *p* < 0.01, respectively), while the second was even more pronounced when follow-up CSF analyses were included (*p* < 0.001). CSF findings help to distinguish TBE from other viral infections. In cases with CSF pleocytosis and a positive history for a stay in or near an endemic area, TBE antibodies in CSF and serum should be determined, especially if granulocytes in CSF cell differentiation and/or an intrathecal IgM synthesis is present.

## 1. Introduction

Tick-borne encephalitis (TBE) is an acute inflammatory disease of the central nervous system (CNS) caused by an RNA flavivirus of the same name (tick-borne encephalitis virus, TBEV) and transmitted by ticks [1]. In Europe, the most common vector for the TBEV is Ixodes ricinus [1]. Usually, TBEV infection has a biphasic course, with a prodromal phase with flu-like symptoms first, followed by a meningitic/encephalitic phase several days later in some patients. Symptoms range from mild meningitis to severe encephalitis with or without myelitis [2]. TBE is endemic in the Eurasian northern hemisphere, with at least 10,000 cases annually [3], yet it is a rare disease. An increasing incidence of TBE despite the availability of effective vaccines has been described, possibly due to an extended season of the infection and the enlargement of endemic areas [4,5].

The standard method to diagnose TBEV infections is the detection of specific IgM and IgG antibodies in serum and/or CSF if there is a positive history for a stay in an endemic area [3]. There is no specific treatment for TBE. In general, patients with CNS infections are empirically treated with intravenous aciclovir and antibiotics until herpes simplex virus (HSV) or other pathogens and autoimmune encephalitis are ruled out. In order to avoid unnecessary treatments with potential side effects, it is important to diagnose TBE despite the lack of causal therapy.

Therefore, we aimed to describe cerebrospinal fluid (CSF) and clinical findings, including magnetic resonance imaging (MRI) data, in TBE in comparison with HSV- and VZV-induced CNS infections (HSV-I, VZV-I), which are the main differential diagnoses.

## 2. Materials and Methods

CSF findings of patients with suspected viral CNS infections who were admitted to the Department of Neurology of the University of Ulm between July 2009 and July 2018 were reviewed retrospectively. 

### 2.1. Inclusion Criteria

Identification of TBE as well as HSV-I and VZV-I was carried out, fulfilling the following criteria: (1) clinical signs of encephalitis and/or meningitis; (2) complete CSF analysis including cell count and cell differentiation, CSF/serum albumin ratio, oligoclonal IgG bands (OCB), lactate, determination of intrathecal IgG, IgA, and IgM synthesis, and confirmed viral etiology by PCR or antibody testing in CSF; (3) hospitalized patients with sufficient documented clinical history; and (4) available brain MRI at the time of lumbar puncture (LP).

Encephalitis was defined according to the criteria of the International Encephalitis Consortium [6]; encephalopathy (altered consciousness that persisted for longer than 24 h, including lethargy, irritability, or a change in personality and behavior) and with ≥2 of the following: fever or history of fever (≥38 °C) during the presenting illness; seizures and/or focal neurological findings; CSF pleocytosis (more than four white blood cells per µL); electroencephalographic (EEG) findings indicative of encephalitis; and/or abnormal results of MRI suggestive of encephalitis.

### 2.2. Ethics

The local ethics committee of the University of Ulm approved this study (ethics approval number 20/10).

### 2.3. Statistics

All statistical analyses were performed using the software IBM SPSS Statistics Version 25. Continuous variables were described by median (first quartile–third quartile) and categorical variables by absolute and relative frequencies. Comparisons between the three groups were performed using Fisher’s exact test for categorical variables and the Kruskal–Wallis test for continuous variables. Post hoc analyses were performed using Fisher’s exact test and Mann–Whitney U test, respectively. Furthermore, we used multiple logistic regression analysis when confounding or effect modification was suspected, yielding odds ratios (ORs) and 95% confidence intervals (CIs). Interaction terms between two variables were calculated by multiplication. We used stepwise variable selection (forward selection based on likelihood ratio) for the identification of relevant independent variables in the regression model. Statistical significance was assumed for *p*-values of less than 0.05. In this explorative analysis, there was no correction of *p*-values for multiple testing. Graphic Program by Albaum IT-Solutions was used to visualize the Reiber diagrams.

### 2.4. Data Availability

Anonymized data on individual patient level will be shared by request from any qualified investigator.

## 3. Results

In total, 59 patients with 118 CSF/serum pairs were included. All patients were hospitalized. MRI scans, LP, and complete CSF analysis were available. Viral etiology was confirmed by PCR in CSF or detection of increased pathogen-specific CSF/serum antibody indices (AIs) (calculated according to [7]).

Twenty-one patients with TBE (35 CSF/serum pairs including follow-up LP), 20 with HSV-I, and 18 patients with VZV-I (40 and 43 CSF/serum pairs, respectively) were identified. For demographic and clinical characteristics, see Table 1.

Sixteen of the 21 TBE patients (76%) were male, 11 (52%) presented with meningitis, and nine (43%) as meningoencephalitis. Three TBE patients (14%) showed leptomeningeal enhancement upon brain MRI. Electroencephalography (EEG) showed general slow activity in all five (24%) studied patients and epileptiform pattern in one TBE patient. Onset of neurological symptoms in TBE was longer at the time of first diagnostic LP as compared with HSV- and VZV-I (*p* < 0.01). When compared with HSV-I, TBE was significantly more often associated with a meningitic than an encephalitic presentation (*p* < 0.01). Inflammatory MRI changes (3/21) were significantly less frequent than in HSV-I (14/20, *p* < 0.001). The diagnosis of viral etiology was confirmed by either elevated virus-specific IgG AI or positive virus PCR. In detail, the virus-specific IgG AI was elevated in all TBE (AI was tested in 20/21 patients in the first LP and was elevated in 17; it was elevated all three previously negative re-tested patients and the one patient not tested upon the first LP), in 85% of HSV-I (AI was tested in 12/20 patients in the first LP and was elevated in six; it was elevated in 5/6 previously negative re-tested patients and six patients not tested upon the first LP), and in 94% of VZV-I (AI was tested in 13/18 patients in the first LP and was elevated in six; it was elevated in all seven previously negative re-tested patients and four patients not tested upon the first LP). CSF virus PCR was positive in the first diagnostic LP in 0 of 9 (0%) TBE, 14 of 17 (82%) HSV-I, and 11 of 13 (85%) tested VZV-I.

**Figure 1:**
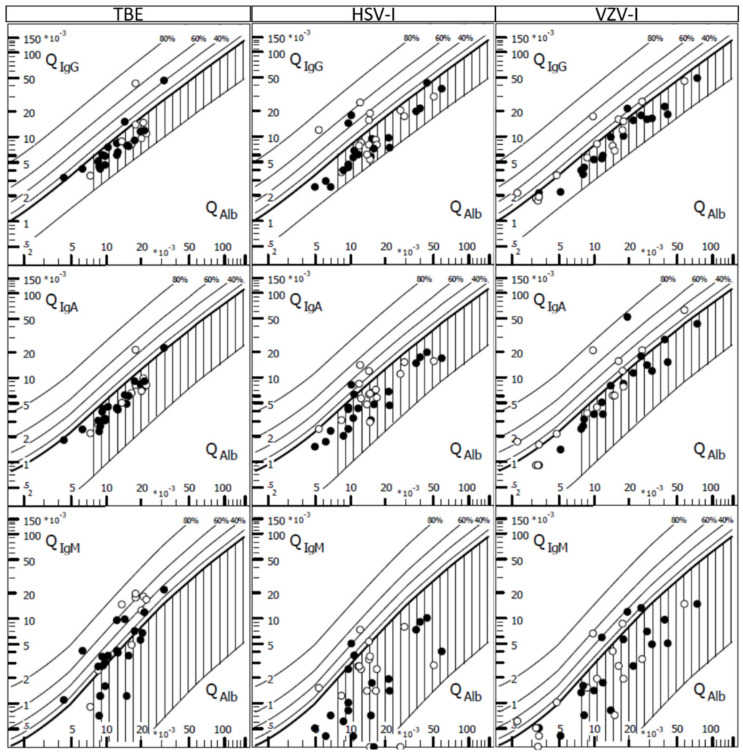
Intrathecal immunoglobulins in tick-borne encephalitis (TBE) and CNS infections by herpes simplex virus (HSV-I) and varicella zoster virus (VZV-I) CSF/serum quotient diagrams for IgG, IgA and IgM with hyperbolic discrimination functions in tick-borne encephalitis (TBE), and CNS infections by herpes simplex virus (HSV-I) and varicella zoster virus (VZV-I). The upper curve of the reference range represents the discrimination line between brain-derived and blood-derived immunoglobulin fractions in the CSF. Filled figures indicate first diagnostic lumbar puncture and open figures indicate one follow-up lumbar puncture. Graphic Program by Albaum IT-Solutions was used to visualize the Reiber diagrams.

CSF analysis at first LP revealed pleocytosis in all patients (Table 2). However, VZV-I had a higher cell count than TBE (*p* = 0.007). In general, the CSF cell differentiation revealed a lymphocytic predominance. A markedly increased proportion of neutrophil granulocytes (>20%) was found significantly more often in TBE (10/21, 48%), compared with 3 (15%) and 1 (6%) patients with HSV-I and VZV-I (*p* < 0.05 and *p* < 0.01), respectively. CSF-specific OCB (3/21, 14%) were found slightly less often in TBE as compared with HSV-I and VZV-I; however, this difference was not significant. An elevated albumin CSF/serum ratio indicating a blood/CSF barrier dysfunction was found in 18 TBE patients (86%), similar to the other two types of CNS infections (Figure 1). Proof of quantitative intrathecal IgM synthesis occurred in 13 (62%) TBE patients, significantly more often that in HSV-I and VZV-I (*p* < 0.001 and *p* = 0.001, respectively) (Figure 1). Upon follow-up LP, proof of quantitative IgM synthesis became apparent in all tested TBE patients. As the duration from symptom onset to LP was longer in TBE as compared with HSV-I and VZV-I (*p* = 0.002 and *p* = 0.005, respectively), we performed a logistic regression (using intrathecal IgM synthesis as the dependent variable) to analyze if this delay was a confounding factor or caused effect modification. However, the final regression model included only the disease/group variables (TBE vs. HSV-I: OR 13.8, 95% CI 2.5 to 76.3, *p* < 0.01; TBE vs. VZV-I: OR 8.1, 95% CI 1.8 to 37.2, *p* < 0.01).

Two case presentations were particularly demanding in terms of diagnosis: In one patient, CSF analysis performed in the first systemic phase of TBE virus (without neurological deficits) infection was completely normal. However, later on, the patient developed encephalitis with prominent pleocytosis and showed positive CSF PCR and antibody testing for TBE in the follow-up lumbar puncture. Another patient, a forester with meningitis, showed both positive TBE and Borrelia antibody indices in the first diagnostic lumbar puncture. However, CSF CXCL13 (a B-cell-attracting chemokine) was, as in 11 other TBE cases in this cohort (*n* = 12, median 32 pg/mL, range 14–240 pg/mL), below the cut-off shown earlier by us to differentiate between neuroborreliosis and other inflammatory CNS diseases [8]. Due to the clinical course (biphasic with a prodromal phase with flu-like symptoms followed by a meningitis some days later, without evidence for encephalopathy or radiculitis), antibiotic treatment was stopped, the patient was discharged with almost no complaints.

## 4. Discussion and Conclusions

Despite an increasing number of TBE cases within the last years, TBE still belongs to the rare diseases. Here, we focused on CSF findings to investigate whether and how CSF findings can contribute to distinguish TBE from HSV-I and VZV-I. Because up to 60% of viral meningitis/encephalitis cases have an unknown cause/pathogen [9,10], we decided to apply strict inclusion criteria with confirmed pathogen detection.

The distribution of the clinical manifestation in our TBE cohort is comparable to earlier reports [1]. Basic CSF variables including leukocyte count and albumin CSF/serum ratio as a marker for blood/CSF barrier function are abnormal in TBE. However, these findings are non-specific and can be found to a similar extent in other viral (e.g., HSV) or subacute bacterial (e.g., borrelia) CNS infections. All patients with TBE showed a CSF pleocytosis, which was less prominent as compared with VZV-I, but not significantly different as compared with HSV-I. The CSF cell differentiation revealed a lymphocytic predominance, as known from other viral CNS infections. In TBE, the substantial presence of granulocytes (>20%) was characteristic, occurring to a larger extent in almost 50% of the cases.

While the sensitivity of an HSV PCR in CSF was described as very high in the first week of the disease [11], TBEV PCR in CSF was not helpful for diagnosis, although only nine cases were tested. This may also be related to a longer duration of symptoms at first diagnostic LP compared with HSV-I. Of more importance is the indirect detection of the pathogen by means of antibody determination in CSF and serum. The pathogen-specific AI proved to be highly valuable, although sometimes only in the course of the disease. In our cohort, 85% (17/20) of tested TBE patients showed an elevated TBE-specific CSF-serum antibody index (AI) already in the first diagnostic LP, indicating intrathecal pathogen-specific antibody production.

Overall, in comparison with VZV-I and HSV-I, the CSF of patients with TBE is characterized by a more frequent intrathecal IgM synthesis and a higher percentage of neutrophil granulocytes upon CSF cell staining. These findings may influence the clinician’s decision in the diagnostic workup (e.g., early testing of TBE AI, increasing the alertness to TBE), but are by no means sufficiently dichotomous to confirm or exclude the TBE diagnosis. However, in the first systemic phase (without neurologic deficits) of TBE virus infection, we show that CSF findings can be normal.

Because TBE and neuroborreliosis are occupational diseases affecting foresters, an elevated borrelia CSF-serum antibody index as a result of a current or previous neuroborreliosis can be expected to occur more often in patients with TBE. Our findings are consistent with previous data suggesting that CSF CXCL13 levels might differentiate between TBE and neuroborreliosis in such cases, because none of the TBE CSF-serum pairs with CXCL13 values available showed levels exceeding the cut-off previously defined by us for neuroborreliosis [8].

In conclusion, in clinically suspected cases, the determination of TBE antibodies in CSF and serum should be performed, especially if granulocytes and an intrathecal IgM synthesis in CSF are present. These findings may contribute to distinguish TBE from other viral CNS infections, as well as in areas where the disease is not yet common, and to avoid unnecessary medication as well as associated side effects.

## Figures and Tables

**Table 1:** Demographic and clinical characteristics.

	TBE	HSV-I	VZV-I	*p*-Value
n (female/male)	21 (5/16)	20 (12/8)	18 (8/10)	0.064
Age (years), median	47 (33–66)	58 (53–71)	59 (34–77)	0.280
Onset of neurological symptoms (days)	7 (5–14)	3 (1–8) *p* = 0.002	4 (2–6) *p* = 0.005	0.002
Meningitis n [%]	11 [52.4]	2 [10.0] *p* = 0.006	10 [55.6] *p* = 1.00	0.004
Encephalitis n [%]	1 [4.8]	13 [65.0] *p* < 0.001	2 [11.1] *p* = 0.586	<0.001
Meningoencephalitis n [%]	9 [42.9]	5 [25.0]	5 [27.8]	0.471
Inflammatory changes in brain MRI n [%]	3 [14.3]	14 [70.0] *p* = < 0.001	2 [11.8] *p* = 1.00	<0.001
Abnormal EEG * n [%]	5 [23.8]	12 [63.2]	6 [33.3]	0.416

Data are given as median (IQR), except when indicated otherwise. EEG, electroencephalography; HSV-I, central nervous system infection by herpes simplex virus; MRI, magnetic resonance imaging; TBE, tick-borne encephalitis; VZV-I, central nervous system infection by varicella zoster virus. * performed in a subset (5 TBE, 16 HSV-I, 9 VZV-I) of patients, when appropriate clinical symptoms were present. Percentages were rounded. *p*-values of paired tests (TBE vs. HSV-I as well as TBE vs. VZV-I) are shown in the corresponding column.

**Table 2:** Cerebrospinal fluid (CSF) findings.

	TBE *n* = 21	HSV-I *n* = 20	VZV-I *n* = 18	*p*-Value
Leucocyte count (/µL) Pleocytosis with >20% granulocytes n [%]	65 (33–120) 10 [47.6]	91 (20–285) *p* = 0.648 3 [15.0] *p* = 0.043	243 (70–302) *p* = 0.007 1 [5.6] *p* = 0.005	0.037 0.007
Q albumin (×10^−3^)	12.6 (9.1–15.9)	11.5 (9.8–21.9)	16.2 (8.4–28.8)	0.540
Total protein	922 (610–1039)	865 (676–1355)	1240 (610–1950)	0.308
Blood-CSF-barrier dysfunction n [%]	18 [85.7]	17 [85.0]	15 [83.3]	1.00
Lactate (mmol/L)	2.0 (1.9–2.2)	2.5 (2.0–3.0)	2.8 (2.3–3.7)	0.010
	*p* = 0.050		*p* = 0.004	
Oligoclonal IgG bands n [%]				
In first LP	3 [14.3]	6 [30.0]	6 [33.3]	0.364
In follow-up LP *	9 [90.0]	12 [80.0]	13 [86.7]	0.871
Intrathecal IgG synthesis n [%]				
In first LP	2 [9.5]	3 [15.0]	1 [5.6]	0.764
In follow-up LP *	2 [20.0]	6 [40.0]	6 [40.0]	0.639
Intrathecal IgA synthesis n [%]				
In first LP	1 [4.8]	2 [10.0]	3 [16.7]	0.420
In follow-up LP *	1 [10.0]	6 [40.0]	8 [53.3]	0.107
Intrathecal IgM synthesis n [%]				
In first LP	13 [61.9]	2 [10.0]	3 [16.7]	0.001
	*p* = 0.001		*p* = 0.008	
In follow-up LP *	10 [100.0]	3 [20.0]	4 [26.7]	<0.001
	*p* < 0.001		*p* = 0.001	

Data are given as median (IQR), except when indicated otherwise. Percentages were rounded. HSV-I, central nervous system infection by herpes simplex virus; LP, lumbar puncture; Q albumin, cerebrospinal fluid to serum albumin concentration ratio; TBE, tick-borne encephalitis; VZV-I, central nervous system infection by varicella zoster virus. *p*-values of post-hoc tests (compared with TBE) are shown in the corresponding column. *p*-values of paired tests (TBE vs. HSV-I as well as TBE vs. VZV-I) are shown in the corresponding column. * at least one follow-up LP was performed in 10 TBE, 15 HSV-I, and 15 VZV-I.
